# Critical Factors for Health Behavior Among University Students: The Role of Health Consciousness, Health Knowledge, and Risk Perception

**DOI:** 10.3390/healthcare14121645

**Published:** 2026-06-10

**Authors:** Qingteng Wei, Yubo Zhou, Zhen Qin, Siu Shing Man, Yao Li, Alan Hoi Shou Chan

**Affiliations:** 1 School of Design, South China University of Technology, Guangzhou 510006, China; 202330741221@mail.scut.edu.cn (Q.W.); qinzhen1988@scut.edu.cn (Z.Q.); ssman6@scut.edu.cn (S.S.M.); 2 School of Computer Science, Beijing Institute of Technology, Beijing 100081, China; 1120231857@bit.edu.cn; 3 College of Engineering, City University of Hong Kong (Dongguan), Dongguan 523808, China; alan.chan@cityu.edu.hk

**Keywords:** health behavior, university student, theory of planned behavior, health consciousness, health knowledge, risk perception

## Abstract

**Background:** In an era of sedentary lifestyles and multifaceted health challenges, adopting health behavior (HB) is essential to address the health problems threatening the physical health of university students. This study aimed to explore the factors associated with HB among university students. The University Student Health Behavior Model (USHBM) was developed based on the theory of planned behavior and incorporated health consciousness (HC), health knowledge (HK), and risk perception (RP). **Methods:** Using a cross-sectional survey design, data were collected from 384 university students in China. Structural equation modeling was employed to evaluate the hypothesized relationships of the USHBM. **Results:** HK significantly and directly predicted behavioral intention (BI) (*β* = 0.421, *p* < 0.001). Perceived behavioral control (PBC) emerged as the strongest associated factor of BI (*β* = 0.417, *p* < 0.001). HC was significantly and positively related to attitude toward behavior (ATT) (*β* = 0.451, *p* < 0.001), subjective norm (SN) (*β* = 0.332, *p* < 0.001), and PBC (*β* = 0.357, *p* < 0.001). Furthermore, RP functioned as an associated factor of HB (*β* = 0.411, *p* < 0.001). Additionally, RP was significantly and positively associated with the adoption of HB. **Conclusions:** The findings provided an in-depth understanding of university students’ HB. According to the findings, practical implications for enhancing university students’ HB were discussed.

## 1. Introduction

University students are increasingly plagued by a multifaceted array of health problems that pose significant threats to their long-term well-being. Daily electronic screen use exceeding eight hours leads to prolonged sedentary behavior among university students, significantly increasing their susceptibility to long-term cardiovascular and metabolic risks [1]. Moreover, over 60% of Chinese university students suffer from imbalanced nutritional intake [2]. This exposure to health risks correlates with a surge in obesity prevalence among Chinese college students, which rose from 3.7% in 1995 to 23.4% in 2019, and is projected to reach 36.0% by 2030 [3]. Furthermore, over 50% of Chinese university students experience poor sleep quality, primarily due to excessive late-night use of digital devices [4]. To address these health problems, adopting healthy behavior (HB) is essential for sustaining long-term wellness among university students.

HB encompasses any activity undertaken by an individual to protect, promote, or maintain health [5]. From a physiological perspective, HB is defined as activities that directly modulate the internal biological environment of an organism. These activities function by regulating critical processes, including metabolic homeostasis, endocrine signaling, and immunological resilience [6]. For instance, consistent engagement in physical exercise, nutritional self-regulation, and restorative sleep is fundamental for maintaining physical and mental health [7,8,9]. Specifically, regular physical exercise establishes a biological framework to counteract cardiovascular and metabolic risks among university students by fostering hippocampal neuroplasticity and promoting cognitive efficiency [10]. Besides, as a fundamental cornerstone of health, effective nutritional self-regulation optimizes tissue function and immunity, maintaining internal balance and preventing obesity-driven chronic diseases [11]. Furthermore, Ahmed [12] emphasizes that restorative sleep is an essential physiological process that helps stabilize university students’ psychological states and systemic functions.

Given the vital role of HB in alleviating university students’ health problems, it is important to explore the factors associated with their HB. The theory of planned behavior (TPB) is frequently utilized as a comprehensive analytical lens because it provides a structured framework for identifying the core determinants of individual actions [13]. Previous research has expanded the TPB framework by integrating a diverse array of variables to predict HB among university students [14,15]. For instance, Wang and Kang [16] integrated sport experience into the TPB to demonstrate that this factor significantly strengthens university students’ behavioral intentions to engage in physical exercise. Similarly, Jooyandeh et al. [17] investigated how risk perception (RP), within an expanded TPB framework, influences Iranian students’ behavioral intentions to utilize probiotic products, thereby facilitating the cultivation of healthy dietary habits. Recent literature has demonstrated that health consciousness (HC) can increase the frequency of sports activities among university students [18]. Additionally, health knowledge (HK) is a fundamental factor of HB among university students [19]. Although the aforementioned studies have enhanced comprehension of university students’ HB, the current literature remains fragmented, leaving a critical research gap unresolved. Specifically, although the literature has established the significance of HC, HK, and RP, their collective association with HB among university students has not been sufficiently examined within an integrated quantitative framework. Consequently, there is a pressing need to investigate how HC, HK, and RP synergistically relate to university students’ HB.

To address this research gap, the present study proposed a theoretical framework integrating HC, HK, and RP into the TPB to account for HB among university students. HC, HK and RP were selected for two reasons. First, HC, HK, and RP have been widely recognized as crucial precursors in predictive HB models [17,20] and public wellness interventions [21]. Second, HC establishes a proactive baseline wellness orientation [22], whereas HK provides the requisite informational foundation [23] and RP supplies the essential self-protective motivation to translate internal beliefs into tangible actions [24]. Besides, the research model of this study improves upon generic TPB-based frameworks by providing a unified structure that specifically targets the HB of university students. The research model resolves the fragmentation in existing literature and offers a robust explanation of how students’ internal consciousness, concrete knowledge, and subjective risk judgments are associated with their HB. The findings can provide an in-depth understanding of university students’ HB. In addition, the findings can provide a theoretical foundation for health practitioners and educators to develop effective interventions that promote HB among university students. Ultimately, university students’ long-term well-being can be enhanced. The following section reviews related work and describes the proposed model’s development process.

## 2. Research Hypotheses

### 2.1. Theory of Planned Behavior

**Figure 1:**
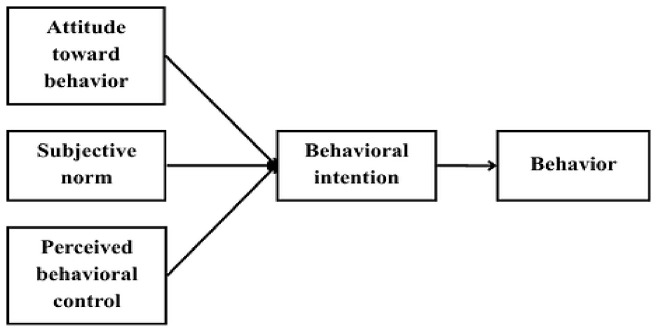
Theory of planned behavior proposed by Ajzen [13].

The TPB (Figure 1) was developed by Ajzen [13] to explain individual behavior. Because the TPB is a generic model, this study extended its original constructs to capture the specific determinants of university students’ HB. In the TPB, three important constructs, namely, attitude toward behavior (ATT), subjective norm (SN), and perceived behavioral control (PBC), are positively related to a behavioral intention (BI), which in turn is associated with the behavior. In this study, ATT is defined as university students’ positive or negative feelings towards engaging in HB. SN refers to the perceived pressure from peers or family to perform HB, whereas PBC is defined as the perceived ease or difficulty of performing HB. BI represents the strength of a student’s intention to adopt HB. The TPB has been widely applied to psychological health [25], road safety [26], and vaccination intention among the public [27]. According to the TPB [13], the following hypotheses were proposed:

**H1.**  *ATT is positively related to BI.*

**H2.**  *SN is positively related to BI.*

**H3.**  *PBC is positively related to BI.*

**H4.**  *BI is positively related to HB.*

### 2.2. Health Consciousness

HC is a cognitive determinant characterized by heightened self-awareness of one’s health status, which is associated with proactive concern and a willingness to adopt health-promoting behaviors [20,28]. Beyond psychological awareness, HC reflects the degree to which individuals integrate health concerns into their daily lives and take personal responsibility for their long-term well-being [29]. Furthermore, highly health-conscious individuals are characterized by an active engagement in seeking health-related information and a consistent motivation to adhere to wellness recommendations [30]. While Li and Shan [31] demonstrated that HC significantly increases the public intention to consume healthy products, this relationship has not been verified in the specific context of university students’ HB. Additionally, McKinley et al. [32] demonstrated that HC positively influences ATT, SN, and PBC in the context of COVID-19 prevention behavior among university students. Following these insights, this study proposed these hypotheses:

**H5.**  *HC is positively related to ATT.*

**H6.**  *HC is positively related to SN.*

**H7.**  *HC is positively related to PBC.*

### 2.3. Health Knowledge

HK is the cognitive capacity for understanding and retaining health-related knowledge, serving as a critical internal resource for facilitating behavioral change [33]. HK serves as a fundamental prerequisite for behavioral change, providing individuals with the necessary informational basis to evaluate the potential benefits and consequences of their health-related actions [34]. Furthermore, HK functions as a multidimensional construct that enables individuals to navigate complex health environments and effectively apply cognitive resources to diverse health challenges [35]. While Rajeh [36] has demonstrated that HK is a significant factor in modeling intentions to improve oral HB, this relationship has not been verified in the specific context of university students’ HB. Given these findings, this study proposed that, within the extended TPB framework, HK is directly associated with university students’ BI. Accordingly, the following hypothesis was put forward:

**H8.**  *HK is positively related to BI.*

### 2.4. Risk Perception

RP refers to people’s subjective judgments about risks [37,38,39]. In this study, RP is defined as the subjective assessment of the likelihood and severity of negative health consequences that university students may experience due to suboptimal lifestyle choices [12,40,41]. Beyond purely cognitive evaluations, RP encompasses a sense of personal vulnerability that transforms abstract health threats into concrete motivations for self-protection [42]. RP is often influenced by affective components, where the emotional weight of potential outcomes dictates the urgency of adopting HB [43]. While recent research demonstrated that RP negatively influences consumers’ purchase behavior regarding suboptimal food [44], this relationship has not been verified in the specific context of university students’ HB. In the context of university life, RP is characterized by a heightened awareness of the potential physiological damage caused by unhealthy routines, which results in a motivation to adopt protective measures [45]. Accordingly, the following hypothesis was put forward:

**H9.**  *RP is positively related to HB.*

### 2.5. Research Model

**Figure 2:**
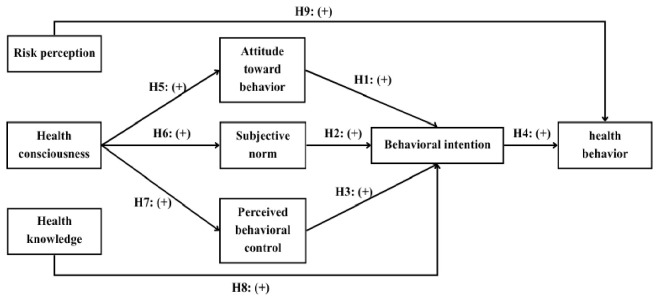
University Student Health Behavior Model.

This study developed a research model, the University Student Health Behavior Model (USHBM), derived from the nine above-mentioned hypotheses related to TPB, HC, HK, and RP, to gain a comprehensive understanding of HB among university students. Figure 2 illustrates the USHBM. HC is a distal and foundational mindset representing a stable dispositional trait and a general awareness of wellness. According to the core tenets of the TPB, broad personality or psychological orientations do not directly dictate specific behaviors [13]. Instead, personality or psychological orientations must be filtered and operationalized through ATT, SN and PBC before they can formulate concrete intentions [31]. HK is a proximal cognitive tool directly shaping intentions and a tool for self-regulation. Within behavior frameworks, the acquisition of knowledge among university students can directly catalyze the formulation of BI [46]. The direct path from RP to HB is theoretically grounded in the Health Belief Model [47]. Within established HB frameworks, cognitive and affective evaluations of health threats function as immediate internal triggers for actions [48]. Consequently, when university students experience a high level of RP regarding their lifestyle choices, this subjective threat assessment is concurrently linked to their self-protective motivation and the adoption of HB. This configuration demonstrated that the USHBM is built upon a highly robust, multi-layered theoretical foundation that integrates the complementary strengths of TPB, rather than an arbitrary empirical arrangement.

## 3. Method

A structured questionnaire was used to collect data to test the hypotheses outlined in Section 2. The following subsections present the details of the measurements, participants, and data analysis.

### 3.1. Measurements

The questionnaire comprised two sections: (a) demographic characteristics such as age, gender, academic year, and academic discipline, and (b) participants’ perceptions of ATT, SN, PBC, BI, HB, HC, HK, and RP. Table 1 shows the item details and the corresponding references. All constructs were rated on a five-point Likert scale from 1 (strongly disagree) to 5 (strongly agree). The items were initially selected from established scales in previous literature. Because these original items were developed in varying contexts, a systematic modification was executed in strict accordance with the cross-cultural and contextual adaptation protocols recommended by Beaton et al. [49] and DeVellis and Thorpe [50]. These items were carefully modified to specifically reflect university students’ proactive practices regarding their physical health within a campus environment. Given that the original scales were developed in English, a standardized translation and back-translation procedure was employed to ensure linguistic equivalence and cultural appropriateness. Two bilingual researchers translated the items from English into Chinese. Subsequently, an independent bilingual expert back-translated the Chinese version into English. Discrepancies were discussed and resolved by the research team to ensure the original meaning was preserved while reading naturally for Chinese students. To establish content validity, the translated items was evaluated by an expert panel consisting of three scholars specializing in public health, psychology, and survey methodology. The panel assessed each item for clarity, relevance, and cultural suitability. Based on their expert feedback, minor adjustments were made to the wording of items to eliminate ambiguity and improve comprehension. Before the formal data collection phase, a pilot test was conducted with a convenience sample of 40 Chinese university students who were subsequently excluded from the main study. Participants completed the survey and evaluated each item using a 4-point rating scale (1 = completely unclear; 4 = completely clear) to assess readability, wording precision, and formatting. The empirical feedback demonstrated highly favorable metrics, with the mean clarity score for all items exceeding 3.5 out of 4. This high quantitative evaluation, complemented by qualitative suggestions, confirmed that the revised scales were thoroughly unambiguous and psychometrically sound [51]. To reduce potential response bias, participants were informed before the survey began that they had the right to withdraw at any time and that all collected data would be processed anonymously and confidentially.

### 3.2. Participants

The study population consisted of students enrolled at universities in China during the study period. Specifically, the data collection period spanned from October 2025 to December 2025, involving participants across five distinct higher education institutions. A convenience sampling method was used to recruit eligible participants. The inclusion criteria were as follows. Students aged 18 years or older, currently registered as full-time university students, able to understand the questionnaire language, and willing to provide informed consent. To ensure targeted data collection, recruitment was conducted through distinct offline and online channels. Offline recruitment took place at physical sites, including campus libraries and student dining halls, via class-based invitations and student organizations. Meanwhile, online recruitment leveraged university forums along with digital platforms widely used by students, such as institutional emails, learning management systems, and social media groups. The recruitment message briefly introduced the study purpose, emphasized voluntary participation and confidentiality, and provided a link or QR code to the questionnaire. Before completing the survey, participants were required to read an information sheet and provide electronic informed consent. To reduce duplicate responses, each participant was allowed to complete the questionnaire only once, and responses were screened for completeness and eligibility before statistical analysis. All participants provided informed consent, and the study was conducted in accordance with the Declaration of Helsinki.

### 3.3. Data Analysis

The USHBM was tested with structural equation modeling (SEM). According to Anderson and Gerbing [56], the measurement model was examined using confirmatory factor analysis to assess its reliability and construct validity. Cronbach’s alpha [57], a measure of internal consistency reliability, was used with a criterion level of 0.7 [58]. Convergent validity evaluates whether indicators designed to represent the same latent construct demonstrate sufficient agreement, whereas discriminant validity examines whether separate constructs are empirically distinct and not excessively correlated [59]. Following the criteria proposed by Fornell and Larcker [60], convergent validity can be supported when each construct demonstrates a composite reliability (CR) above 0.70 and its measurement items exhibit factor loadings greater than 0.70. Furthermore, an AVE value above 0.50 was required for each construct. The discriminant validity of the measurement model was evaluated according to the Fornell–Larcker approach [56]. Specifically, the square root of the AVE for each construct was compared with the correlations between that construct and the remaining constructs. Discriminant validity was considered acceptable when the square root of the AVE was higher than the corresponding inter-construct correlations. Subsequently, structural equation modeling (SEM) was applied to estimate the structural paths and test the proposed hypotheses. To evaluate the construct validity and suitability of the data, the measurement and structural models were comprehensively assessed using key global model-fit indices. Model fit indices, namely, the ratio of chi-square to its degree of freedom (*χ*^2^/*df*), root mean square error of approximation (RMSEA), standardized root mean squared residual (SRMR), Tucker–Lewis index (TLI), and comparative fit index (CFI), were used to assess the measurement models [61]. The criteria of the model fit indices included *χ*^2^/*df* < 5 [62], TLI ≥ 0.95, CFI ≥ 0.95, SRMR < 0.08 [63], and RMSEA < 0.08 [64]. All statistical analyses were performed using SPSS 21 and AMOS 21.

## 4. Results

### 4.1. Participant Characteristics

A total of 412 university students participated in the study. Following the screening process, a final sample size of 384 valid responses was retained, yielding a valid response rate of 93.2%. According to the parameter-to-sample heuristic recommended by Kline [65] and the standard 10-times rule [66], the USHBM estimating 24 observational variables necessitated a minimum of 240 active respondents to guarantee stable path coefficients and reliable model-fit indices. Thus, the current sample size of 384 strictly satisfied and exceeded this rigorous econometric threshold, ensuring adequate statistical power for the USHBM validation. Among the participants, 165 were male, and 219 were female, accounting for 43.0% and 57.0% of the sample, respectively. The mean age of the participants was 20.8 years, with a standard deviation of 2.1 years. Participants were distributed across different academic years and academic disciplines. In terms of academic level, most participants were undergraduate students, with 276 students accounting for 71.9% of the total sample, while 108 students were postgraduate students, accounting for 28.1%. Among the undergraduate students, 78 were first-year students, 72 were second-year students, 66 were third-year students, and 60 were fourth-year students. Regarding academic disciplines, 116 students were from medicine-related majors, accounting for 30.2%; 104 were from science and engineering, accounting for 27.1%; 82 were from humanities, accounting for 21.4%; and 82 were from social sciences, accounting for 21.4%. Overall, the sample included students from different academic levels and disciplines, providing a diverse representation of university students.

### 4.2. Measurement Model Assessment

As shown in Table 2, the measurement model yielded an acceptable global fit to the empirical data: *χ*^2^/*df* = 2.395, TLI = 0.961, CFI = 0.972, RMSEA = 0.060, and SRMR = 0.045. All fit indices fully satisfied the designated criteria, demonstrating high construct validity. As shown in Table 3, Cronbach’s alpha values for all constructs ranged from 0.844 to 0.918, exceeding the recommended threshold of 0.70. This indicates that the measurement model demonstrated satisfactory internal consistency reliability. The results in Table 3 also confirmed that the required criteria for convergent validity were met. In addition, the inter-construct correlations were lower than the square roots of the corresponding AVE values, as presented in Table 4, providing evidence of acceptable discriminant validity. Given that the data for all constructs were collected from university students at a single point in time using a self-reported questionnaire, potential common method variance was evaluated. Harman’s single-factor test was conducted by loading all 24 observational items into an unrotated exploratory factor analysis. The results indicated that the first principal factor accounted for only 28.4% of the total variance, which is well below the conservative academic threshold of 50% [67]. This empirical evidence demonstrated that no single dominant factor emerged to explain the majority of the covariance, confirming that common method variance did not pose a significant threat to the validity of the hypothesized structural paths in this study.

### 4.3. Structural Model Assessment

**Figure 3:**
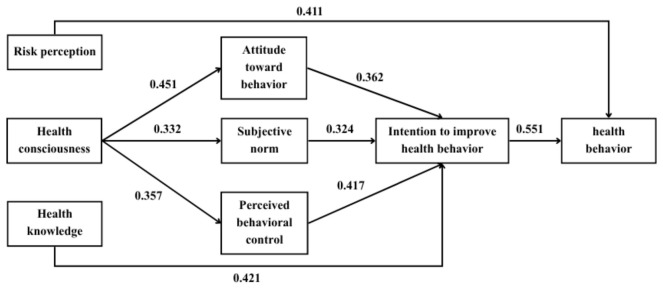
USHBM results.

The structural model (USHBM) was analyzed. The global fit statistics for the structural model also exhibited an adequate fit (*χ*^2^/*df* = 2.490, TLI = 0.956, CFI = 0.969, RMSEA = 0.062, and SRMR = 0.051). Nine hypotheses were supported (Table 5). ATT, SN, and PBC were positively related to BI. BI was significantly and positively related to HB. HC was positively associated with ATT, SN, and PBC. HK was significantly and positively associated with BI. RP was positively associated with HB. These results are presented in Figure 3.

## 5. Discussion

This study developed and empirically supported the USHBM model to address the research gaps identified in the previous literature. By extending the TPB to include HC, HK, and RP, this study developed a framework to account for the factors associated with university students’ HB. The findings provided a foundation for health practitioners and educators to develop strategies to promote HB among university students. The subsequent sections present the theoretical contributions, practical implications, and limitations of this study.

### 5.1. Theoretical Contributions

This study contributed to the relevant body of literature in several important theoretical ways. First, the applicability of the TPB to the explanation of university students’ HB was supported. In this study, BI was positively associated with ATT, SN, and PBC. This result was consistent with the theory of planned behavior [13], which posits that ATT, SN, and PBC are important factors associated with people’s intention to engage in the behavior. Additionally, the results confirmed that BI was significantly positively related to HB. Among these three primary factors (ATT, SN, and PBC), PBC was found to have the strongest association with HB among university students, followed by ATT. These results are consistent with findings in other behavioral research contexts, such as consumer behavior [68] and construction safety [69].

The current research revealed that HC showed significant positive associations with ATT, SN, and PBC among university students. These results were in line with the findings of Liang et al. [70]. They reported that HC was positively related to ATT, SN, and PBC within an expanded TPB framework in the context of green furniture consumption among general consumers. Similarly, Li and Shan [31] highlighted the importance of HC in shaping university students’ psychological predispositions toward healthy lifestyles. University students with high HC exhibit acute self-awareness, enabling them to perceive health-promoting behaviors as rewarding [71], thereby fostering a favorable attitude toward health activities. University students’ sense of personal responsibility for long-term well-being also makes them receptive to the social support and health-related expectations of family and peers, which reinforces the SN they feel within their social circles [72]. The tendency of health-conscious students to actively seek health information enhances their PBC by providing the confidence and resourcefulness needed to effectively manage health challenges within the campus environment [73]. Additionally, the present study uncovered a systematic pathway where HC facilitates the adoption of HB by relating to BI through the mediating mechanisms of ATT, SN, and PBC. These findings empirically supported the research of Terry and Branham [74]. They demonstrated that structured behavior modification interventions can significantly enhance students’ self-efficacy, thereby facilitating the transition of HC into health lifestyle changes. This finding indicated that when university students make a conscious effort to monitor their health, they are likely to successfully internalize health-promoting activities into their daily lives. This internal orientation ensures that proactive health remains a sustained priority, fostering an enduring health culture by establishing long-term motivational mechanisms within university students’ cognitive processes. It is critical to recognize that a high level of HC among university students might not stem entirely from intrinsic wellness values, but could instead be associated with external social media pressures or body image anxieties [32,75]. Such trend-associated awareness often fosters superficial, short-term behavioral compliance rather than a deeply internalized commitment to HB. Consequently, future research should distinguish authentic HC from anxiety-associated health obsessions to avoid overestimating its efficacy in promoting long-term HB among university students. By evaluating HC alongside the TPB constructs, this research offered deep insights into the internal mechanisms that translate university student’s HC into HB.

HK showed a significant positive association with BI of university students. This finding supported the findings of Rajeh [36] that HK is a significant factors of the intention to improve university students’ HB. Similarly, Alp and Akbulut [76] demonstrated that HK functions as a critical cognitive tool that strengthens university students’ self-control, thereby driving the formation of HB. The psychological mechanisms through which HK is related to BI of university students are elaborated as follows. First, HK provides the cognitive foundation that enables university students to evaluate the benefits and consequences of their behavior. By acquiring nutrition knowledge, university students are empowered to manage their diet independently, enabling them to establish healthy eating patterns during their university life [34]. Second, HK functions as a critical cognitive tool for self-regulation. University students with high HK can use health-related information to avoid health-risk behaviors, thereby strengthening their intention to adopt HB [21]. Rather than operating as a completely uniform psychological factor, HK often fails to promote BI due to the persistent knowledge-behavior gap [77]. Specifically, when encountering complex health guidelines, psychological barriers such as uncertainty and hot cognition can prompt students to emotionally dismiss or rationalize away their suboptimal lifestyle choices. Therefore, a simple linear model overlooked environmental barriers. In reality, concrete campus constraints, such as rigid schedules and limited institutional support, were what truly dictated whether a student intended to act. By confirming that HK is directly related to the BI among university students, the current study provided a comprehensive explanation of how HK empowers them to achieve long-term well-being.

RP was significantly and positively associated with HB adoption. This result was in line with the findings of Zhang et al. [78]. They reported that RP had a significant positive effect on HB adoption among patients with recurrent ischemic stroke in China. RP plays an important role in human self-protection behavior [79]. The following two dimensions can explain the relationship between RP and HB. First, RP helps university students realize the risks posed by unhealthy conditions. By recognizing their susceptibility to infection, university students are inclined to adopt HB to safeguard their long-term health [80]. Second, the adoption of HB is substantially associated with affective risk appraisal rather than purely rational, cognitive logic. For instance, university students might initiate a structured physical exercise program not purely due to objective cognitive awareness, but because of heightened perceived susceptibility to adverse physiological transformations. This emotional weight, often characterized by worry regarding the perceived severity of health deterioration such as obesity, frequently dictates the urgency of behavioral modification more effectively than objective clinical assessments alone [42]. Consequently, when university students acknowledged that they were facing health risks, it generates a robust self-protective motivation that was associated with their inclination toward practical interventions. Conversely, while RP was shown to be positively related to HB, excessive RP can backfire, triggering defensive denial among university students. When faced with severe long-term threats such as chronic sleep deprivation or metabolic diseases, university students who lack sufficient immediate coping resources or self-efficacy may choose to psychologically ignore the danger to alleviate immediate anxiety [78]. Thus, the observed positive relationship may only exist within an optimal psychological threshold, beyond which heightened RP paralyzes rather than motivates proactive health choices in university students.

### 5.2. Practical Implications

Based on the findings, four practical recommendations were proposed to encourage university students to adopt HB. First, because PBC showed a strong association with BI, greater emphasis should be placed on improving students’ actual capacity to engage in HB by reducing environmental and resource barriers. Accordingly, accessible physical activity opportunities, extended access to sports facilities, affordable healthy food options, sleep hygiene support, self-monitoring tools, counselling resources, and supportive campus environments were recommended. Second, because HK was positively associated with BI, it was important to focus on health education, such as clear nutritional information, practical guidance on diet, sleep, and exercise, and skills-based health workshops. Third, because HC was associated with BI through ATT, SN, and PBC, peer-supported behavior-change programmes and campus-level health promotion strategies should be developed to strengthen university students’ positive attitudes and supportive social norms. Finally, because RP was associated with HB, it was clarified that risk-oriented interventions, including interactive or simulation-based approaches, should be used selectively to make health risks concrete rather than as standalone interventions. University health programs could explore the integration of virtual reality (VR) simulations into their wellness curricula [81]. By enabling university students to see and feel these potential health threats in a simulated environment, VR transforms abstract health warnings into vivid experiences [82]. Such technology may effectively prompt students’ affective risk appraisals and health concerns.

### 5.3. Research Limitations

Although this study offered meaningful theoretical and practical contributions, six limitations should be acknowledged. First, although the proposed model was developed based on the TPB and relevant HB literature, the present cross-sectional design cannot establish temporal precedence or rule out reverse causality. It is possible that students who already engage in HB, or who perceive themselves as healthy, may report high levels of HC, HK, PBC, and BI. Future studies should use longitudinal designs to establish the temporal ordering among HC, HK, RP, and TPB-related constructs, thereby reducing concerns about reverse causality [3]. Second, given that data collection was limited to China, cultural differences may restrict the extent to which the findings can be generalized to other national or regional contexts. Replication studies in different cultural settings are therefore needed to further evaluate the generalizability of the results. Third, the operationalization of HB in this study primarily captured university students’ self-reported general orientation toward health, rather than concrete and observable lifestyle practices. While the current items effectively measured individuals’ subjective commitment to HB, they did not assess specific metrics such as the frequency of physical activity, exact sleep regularity, or dietary patterns. Future research should strengthen the measurement framework by incorporating validated, domain-specific indicators to track the actual frequency and execution of distinct HB. Fourth, the use of self-reported measures may introduce consistency bias and social desirability bias. In particular, students may over-report HB or provide internally consistent responses based on their general self-perception as healthy individuals. Future studies should incorporate objective or multi-source measures of HB, such as wearable-device data, physical activity records, sleep logs, dietary records, or peer/teacher assessments, to reduce recall bias and social desirability bias [45]. Fifth, the utilization of convenience sampling may introduce inherent self-selection bias and result in limited institutional and regional representativeness across the university students. Future studies should employ stratified probability sampling and multi-institutional longitudinal designs across diverse geographic regions to enhance the generalizability of the findings [3]. Sixth, the current measurement of HB remained overly general and lacked the integration of potentially covariates, such as socioeconomic status, body mass index, prior health status, academic pressure, mental health symptoms, access to physical activity, and sleep conditions. Therefore, future investigations ought to deploy multidimensional behavioral scales and explicitly control for diverse sociodemographic, physical, and psychological covariates using advanced multivariable modeling to improve model robustness [8].

## 6. Conclusions

University students do not consistently adopt HB, which is a major factor associated with various physical and mental health problems. However, little was known about the factors associated with HB among university students in the literature. Therefore, this study examined university students’ HB by incorporating the TPB and three critical internal cognitive precursors: HC, HK, and RP. The results supported the applicability of the TPB in understanding the psychological mechanisms behind university students’ health-related intentions. This study also emphasized the importance of HC, HK, and RP in co-varying with ATT, PBC, and BI to adopt HB. The findings provided practical guidance for health practitioners and university authorities in designing evidence-based interventions, institutional wellness policies, and user-friendly digital health platforms that foster HB among university students and contributed to improved health and well-being.

## Figures and Tables

**Table 1:** Questionnaire items.

Construct	Item	Content	Reference
ATT	ATT1	I believe that health behavior can bring long-term benefits.	[52]
	ATT2	Maintaining a healthy lifestyle is worth the time and effort.	
	ATT3	I have a positive attitude toward health behavior.	
SN	SN1	The people in my life (e.g., family/friends) would be in favor of my practicing healthy behavior.	[53]
	SN2	The people in my life (e.g., family/friends) would support me in maintaining my health behavior.	
	SN3	The people in my life (e.g., family/friends) would encourage me to stick to my health behavior.	
PBC	PBC1	Whether or not I engage in health behavior is under my control.	[52]
	PBC2	I believe I can engage in health behavior.	
	PBC3	I feel confident in maintaining health behavior.	
BI	BI1	I plan to engage in health behavior in the coming months, such as actively managing my diet, sleep, and emotions.	[52]
	BI2	I plan to undergo regular health check-ups to monitor my health status.	
	BI3	I intend to set health goals and work towards achieving them.	
HC	HC1	I am very concerned about my physical health.	[31]
	HC2	I often think about health issues.	
	HC3	I actively practice health behavior in my daily life.	
HK	HK1	I have a good grasp of the basic knowledge needed to maintain health behavior.	[36]
	HK2	I am well aware of how practicing different health behavior affects my body’s long-term well-being.	
	HK3	I know exactly what specific health behavior I should adopt to keep myself in good shape.	
RP	RP1	If I continue with my current lifestyle, I think the chance of developing a health problem in the future is high.	[54]
	RP2	I feel that I am personally vulnerable to the health risks associated with my current daily habits.	
	RP3	I am worried about the potential negative health consequences of my current lifestyle choices.	
HB	HB1	I consistently engage in health behavior in my daily life.	[55]
	HB2	Adopting and maintaining health behavior is one of the best decisions I have made for my long-term health.	
	HB3	Engaging in health behavior remains my long-term health decision.	

**Table 2:** Model assessment results.

Models	*χ*^2^ (*p*-Value)	*χ*^2^/*df*	TLI	CFI	SRMR	RMSEA
Measurement Model	536.418 (*p* < 0.001)	2.395	0.961	0.972	0.045	0.060
Structural Model	572.804 (*p* < 0.001)	2.490	0.956	0.969	0.051	0.062

**Table 3:** Summary of reliability and convergent validity assessment.

Construct	Item	Mean	SD	Factor Loading	AVE	CR	Cronbach’s Alpha
Attitude toward a behavior (ATT)	ATT1	4.001	0.794	0.795	0.659	0.853	0.851
	ATT2	3.957	0.804	0.830			
	ATT3	4.201	0.786	0.810			
Subjective norm (SN)	SN1	4.158	0.776	0.802	0.655	0.851	0.849
	SN2	4.099	0.810	0.844			
	SN3	4.234	0.790	0.781			
Perceived behavioral control (PBC)	PBC1	4.198	0.753	0.768	0.650	0.848	0.846
	PBC2	4.132	0.781	0.832			
	PBC3	4.162	0.811	0.817			
Behavioral intention (BI)	BI1	4.066	0.799	0.808	0.657	0.852	0.851
	BI2	3.989	0.830	0.835			
	BI3	4.009	0.800	0.788			
Health consciousness (HC)	HC1	4.730	0.822	0.844	0.649	0.847	0.846
	HC2	4.068	0.815	0.792			
	HC3	4.245	0.841	0.779			
Health knowledge (HK)	HK1	4.543	0.790	0.814	0.647	0.846	0.844
	HK2	4.696	0.853	0.794			
	HK3	4.385	0.856	0.805			
Risk perception (RP)	RP1	4.108	0.583	0.889	0.794	0.920	0.918
	RP2	4.635	0.924	0.927			
	RP3	4.495	0.299	0.856			
Health behavior (HB)	HB1	4.129	0.935	0.733	0.691	0.870	0.869
	HB2	4.003	0.882	0.892			
	HB3	4.207	0.842	0.861			

**Table 4:** Correlations among constructs.

	ATT	SN	PBC	BI	HC	HK	RP	HB
ATT	0.812							
SN	0.542	0.809						
PBC	0.518	0.476	0.806					
BI	0.505	0.551	0.420	0.811				
HC	0.525	0.447	0.551	0.464	0.805			
HK	0.482	0.437	0.452	0.544	0.493	0.804		
RP	0.491	0.515	0.484	0.544	0.544	0.543	0.891	
HB	0.543	0.528	0.617	0.533	0.543	0.491	0.461	0.832

Note: The diagonal values are square roots of AVE; the off-diagonal values are correlations; ATT = attitude toward a behavior; SN = subjective norm; PBC = perceived behavioral control; BI = behavior intention; HC = health consciousness; HK = health knowledge; RP = risk perception; HB = health behavior.

**Table 5:** Hypothesis testing results.

Hypothesis	Standardized Path Coefficient	*p*-Value
H1: ATT is positively related to BI.	0.362	<0.001
H2: SN is positively related to BI.	0.324	<0.001
H3: PBC is positively related to BI.	0.417	<0.001
H4: BI is positively related to HB.	0.551	<0.001
H5: HC is positively related to ATT.	0.451	<0.001
H6: HC is positively related to SN.	0.332	<0.001
H7: HC is positively related to PBC.	0.357	<0.001
H8: HK is positively related to BI.	0.421	<0.001
H9: RP is positively related to HB.	0.411	<0.001

## Data Availability

The data presented in this study are available upon request from the corresponding author. The data are not publicly available due to privacy restrictions.

## Abbreviations

The following abbreviations are used in this manuscript:

ATT	Attitude toward behavior
AVE	Average variance extracted
BI	Behavioral intention
CR	Composite reliability
HB	Health behavior
HC	Health consciousness
HK	Health knowledge
PBC	Perceived behavioral control
RP	Risk perception
SEM	Structural equation modeling
SN	Subjective norm
TPB	Theory of planned behavior
USHBM	University Student Health Behavior Model
VR	Virtual reality
